# Risk of venous thromboembolism in people with lung cancer: a cohort study using linked UK healthcare data

**DOI:** 10.1038/bjc.2016.143

**Published:** 2016-06-02

**Authors:** Alex J Walker, David R Baldwin, Tim R Card, Helen A Powell, Richard B Hubbard, Matthew J Grainge

**Affiliations:** 1 School of Life Sciences, University of Nottingham, West Block, A Floor, Queens Medical Centre, Nottingham NG7 2UH, UK; 2 Division of Epidemiology and Public Health, School of Medicine, University of Nottingham, Nottingham, UK; 3 Department of Respiratory Medicine, Nottingham University Hospitals, Nottingham, UK; 4 Respiratory Medicine Biomedical Research Unit, Nottingham City Hospital, Nottingham, UK

**Keywords:** venous thromboembolism, lung cancer, pulmonary embolism, deep vein thrombosis

## Abstract

**Background:**

Venous thromboembolism (VTE) is a potentially preventable cause of death in people with lung cancer. Identification of those most at risk and high-risk periods may provide the opportunity for better targeted intervention.

**Methods:**

We conducted a cohort study using the Clinical Practice Research Datalink linked to Hospital Episode Statistics and Cancer Registry data. Our cohort comprises 10 598 people with lung cancer diagnosed between 1997 and 2006 with follow-up continuing to the end of 2010. Cox regression analysis was performed to determine which demographic, tumour and treatment-related factors (time-varying effects of chemotherapy and surgery) independently affected VTE risk. We also determined the effect of a VTE diagnosis on the survival of people with lung cancer.

**Results:**

People with lung cancer had an overall VTE incidence of 39.2 per 1000 person-years (95% confidence interval (CI), 35.4–43.5), though rates varied depending on the patient group and treatment course. Independent factors associated with increased VTE risk were metastatic disease (hazard ratio (HR)=1.9, CI 1.2–3.0 *vs* local disease); adenocarcinoma subtype (HR=2.0, CI 1.5–2.7, *vs* squamous cell; chemotherapy administration (HR=2.1, CI 1.4–3.0 *vs* outside chemotherapy courses); and diagnosis via emergency hospital admission (HR=1.7, CI 1.2–2.3 *vs* other routes to diagnosis). Patients with VTE had an approximately 50% higher risk of mortality than those without VTE.

**Conclusions:**

People with lung cancer have especially high risk of VTE if they have advanced disease, adenocarcinoma or are undergoing chemotherapy. The presence of VTE is an independent risk factor for death.

Lung cancer accounts for 20% of all cancer-related venous thromboembolism (VTE) (Levitan *et al*, 1999; Blom *et al*, 2006; Walker *et al*, 2012), and is associated with a higher incidence of VTE than the average for all cancer patients (Horsted *et al*, 2012). Venous thromboembolism can adversely affect survival in lung cancer patients (Blom *et al*, 2004; Huang *et al*, 2012). Since only a small fraction of lung cancer deaths might be attributable directly to VTE events (Dentali *et al*, 2008), it is possible that the occurrence of VTE often reflects the underlying aggressiveness of the cancer. However, VTE still may cause additional morbidity and disrupt treatment (Kuderer *et al*, 2009).

Clinical trials demonstrate that VTE can be substantially reduced by administration of prophylactic low molecular weight heparin (LMWH) (Verso *et al*, 2010; Agnelli *et al*, 2012), but this may not lead to improvement of survival, as observed in the FRAGMATIC trial (Griffiths *et al*, 2009; Noble, 2014) It may be that thromboprophylaxis needs to be better targeted to confer a survival benefit. Current guidelines (Streiff *et al*, 2011; Kahn *et al*, 2012; Lyman *et al*, 2013) indicate that some cancer patients would benefit from this intervention, including those receiving day case delivered chemotherapy. In addition, it is important for physicians to know which patients are at risk of VTE, so that patients developing VTE can be diagnosed and treated quickly.

Previous studies have identified various high-risk groups for VTE in lung cancer patients, including those with late-stage cancer (Tagalakis *et al*, 2007; Chew *et al*, 2008; Kadlec *et al*, 2014), adenocarcinoma (Blom *et al*, 2004; Tagalakis *et al*, 2007; Chew *et al*, 2008; Kadlec *et al*, 2014), surgery (Connolly *et al*, 2012), chemotherapy (Blom *et al*, 2004; Numico *et al*, 2005; Connolly *et al*, 2012; Kadlec *et al*, 2014) and high platelet count (Zecchina *et al*, 2007). However, none of these studies have been detailed enough to assess their relative importance and ensure their effects are independent. Recent linkage of four UK healthcare databases enables us to study the majority of suspected risk factors for VTE in lung cancer patients. This study uses these databases to determine the precise rates of VTE in lung cancer patients according to tumour type and stage, treatment and other potential risk factors, including age, body mass index (BMI) and pre-existing comorbidity.

## Materials and methods

### Patients and data sources

Our cohort comprises data from four linked healthcare sources: The Clinical Practice Research Datalink (CPRD), Hospital Episodes Statistics (HES), the National Cancer Data Repository (NCDR) and Office for National Statistics (ONS) death certificate data. The present analysis uses patients from approximately 50% of CPRD practices in England, for whom linked data are available.

We selected all patients who had a first lung cancer diagnosis (ICD-10 code C50) between 1 April 1997 and 31 December 2006 (the period from which cancer registry data linked to the CPRD were available). Patients were followed up until they developed a VTE event, died, left a participating GP practice or 31 December 2010, whichever was earliest. Date of cancer diagnosis was the earliest recorded cancer registry date. Patients were excluded for the following reasons:

- If they were under 18 years.
- If they were not in a linked general practice.
- If they were diagnosed with lung cancer outside the CPRD and HES registration period.
- If they were diagnosed in the first year of registration at a participating practice.
- If they had a VTE before first cancer diagnosis.

### Exposures

Cancer stage, pathological type and grade were obtained from cancer registry data. Where known, we classified stage as ‘local disease' (confined to the lung), ‘regional disease' (any lymph-node involvement) or ‘distant metastases'. Route of diagnosis was taken from the admission method of the closest hospitalisation event to the cancer diagnosis date, assuming it occurred within 1 month of diagnosis. Cancer treatments were defined by an OPCS-4 code in the HES data, with additional treatment data obtained from cancer registry data. Surgery codes were specific to procedures used in the treatment of lung cancer. To ascertain radiotherapy treatments, we used cancer registry data exclusively, as radiotherapy is under recorded in HES data (NCIN, 2012). Chemotherapy events recorded within 28 days of each other were grouped together to determine the treatment periods. Patients recorded as having chemotherapy in the cancer registry data, but without a corresponding in-patient record, were assumed to have had outpatient chemotherapy and were included in a separate group, as their timing of treatment could not be determined. Smoking status and BMI were determined from GP records using the latest recording before cancer diagnosis. GP records were also used to calculate Charlson comorbidity score (Charlson *et al*, 1987) (grouped as 0, 1–3 and 4+, with cancer excluded). Platelet count was determined from GP test records, with repeated tests incorporated in a time-varying manner.

### Outcome

A VTE event was confirmed when a relevant medical code in either the CPRD and HES was supported by an anticoagulant prescription or medical code providing evidence of anticoagulation, between 15 days before and 90 days after the VTE event date, or if death occurred within 30 days of the event. Additionally, an underlying cause of death of VTE was included as evidence of a valid VTE event. Only the first VTE event following the cancer diagnosis was considered. This algorithm for defining VTE has been previously validated (Lawrenson *et al*, 2000).

### Statistical methods

Person-time commenced at the time of lung cancer diagnosis, unless a patient had surgery in the 90 days preceding diagnosis, where follow-up started from surgery date. Person-time ended when an outcome (VTE or death) was experienced, or when patients left a contributing general practice. Absolute rates of VTE (uniformly expressed per 1000 person-years) were calculated for all patients and then separately for exposure categories. A Cox proportional hazards model was created to incorporate all the measured exposures. BMI, comorbidity, pathological type, route of diagnosis, cancer stage, cancer grade and radiotherapy treatment were all time-independent (fixed-time) covariates, while other cancer treatments (surgery and chemotherapy) and platelet count were allowed to vary over time. For surgery and chemotherapy, we measured VTE risk (i) before treatment, (ii) during treatment (chemotherapy) and (iii) in monthly periods post treatment. Chemotherapy not recorded in in-patient HES, but recorded in cancer registry data which was measured in a time-independent manner, as treatments in the cancer registry are recorded in as a binary variable, meaning that the time of therapy could not be ascertained. Platelet count was categorised into ‘low' (<140 × 10^6^ ml^−1^), ‘normal range' (140–400 × 10^6^ ml^−1^) and ‘high' (>400 × 10^6^ ml^−1^).

A survival analysis was performed to determine the risk of death following VTE. To eliminate the likelihood of immortal time bias (whereby patients in the VTE group appear to survive for longer due to them having survived long enough to be diagnosed with VTE), we defined VTE as a time-varying covariate, where patients started in the ‘No VTE' group and were switched to the ‘VTE' group at the date of VTE diagnosis. All data management and statistical analysis were performed using Stata version 11 (Statacorp, College Station, TX, USA).

This study was approved by the Independent Scientific Advisory Committee, protocol number- 10_091.

## Results

### Patient characteristics

A total of 10 598 people were diagnosed with lung cancer between 1997 and 2006 (Table 1). Median age at cancer diagnosis was 72 years (IQR, 64–79 years). Among patients whose cancer pathology was recorded (63.0%), small cell made up 19.4% of the population, while for non-small cell patients, squamous cell was the most commonly occurring subtype (37.9%), followed by adenocarcinoma (24.5%). Stage recording in these databases only occurred in 29.7% of cases. Of these, almost two-thirds (62.5%) were recorded as having metastatic disease.

Only 10.8% of the sample underwent surgery (29.9% local/regional, 2.9% metastatic), while 24.9% underwent chemotherapy. First surgery occurred on average 12 days (IQR, 0–54) after the recorded cancer diagnosis; among patients who had both chemotherapy and surgery, the first record of chemotherapy was an average of 69 days (IQR 24–387 days) after surgery.

**Figure 1:**
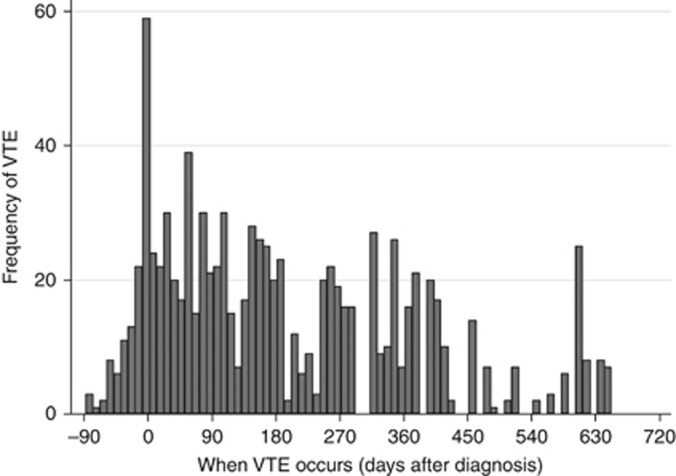
Histogram to describe the frequency of VTE occurrence over the first 2 years after diagnosis (time 0 is the date of diagnosis).

Diagnosis of VTE was recorded in 364 cases among 9284 person-years of follow-up, corresponding to a rate of 39.2 per 1000 person-years (95% confidence Interval, CI, 35.4–43.5). This rate was 11.8 times (95% CI, 10.6–13.1) higher than in age-matched controls as shown in our previous paper from this cohort (Walker *et al*, 2012). The median time to VTE diagnosis was 107 days (IQR 37–336 days). Full characteristics of patients can be found in Table 1. Figure 1 describes when VTE occurred in this population. Here, it is evident that the risk of VTE is mostly clustered around the time of diagnosis, with risk slowly declining subsequently. The risk of VTE in the first 6 months following diagnosis was 76.7 per 1000 person-years (CI 67.5–87.2), declining to 35.6 (CI 27.3–46.3) in the following 6 months, and 15.8 (CI 12.6–19.9) beyond 1 year from diagnosis.

### Patient and tumour-related factors and risk of VTE

The best non-treatment predictors of VTE were histology, cancer stage and diagnosis route, while cancer grade, pre-existing comorbidity, BMI, age and smoking were less strong predictors, or did not affect VTE risk (Table 2).

The multivariable model revealed those with adenocarcinoma had higher risk of VTE than squamous cell patients (hazard ratio (HR)=1.9, CI 1.4–2.6). Patients with distant metastases had a high absolute rate of VTE (81.7, CI 65.0–102.8), but the effect size of stage was reduced in the multivariable model. The variables with the greatest confounding effect were surgery and chemotherapy. However, distant metastases still double the risk compared with local disease (HR=1.8, CI 1.1–2.9). Patients with higher tumour grade had a non-significantly elevated rate of VTE.

Body mass index had little effect on VTE, while there was a trend towards lower risk in older patients (*P*=0.016). Patients who smoke had a higher absolute rate of VTE than those who did not (univariate HR=1.4, CI 1.1–1.7), but this effect was removed after adjustment in the multivariable model (HR=1.2, CI 0.9–1.5). Both pre-existing comorbidity and platelet count had no significant effect on VTE risk.

### Treatment-related factors and risk of VTE

We assessed the effect of different treatment routes on VTE risk, with route of diagnosis and chemotherapy being the strongest predictors of risk.

#### Diagnosis route

Patients diagnosed via emergency hospitalisation had substantially higher rates of VTE than those diagnosed through elective hospitalisation. The rate of VTE for emergency hospitalisation was 71.8 per 1000 person-years (CI 59.4–86.9) *vs* 33.8 (CI 29.6–38.4) for non-emergency admissions. This elevated risk remained evident in the multivariable Cox model, with an HR of 1.6 (CI 1.3–2.1).

#### Surgery

Despite some elevated rates in patients after surgery, none of these reached statistical significance in comparison with patients who did not have surgery. However, it is notable that VTE rate in surgery patients fell substantially below that of non-surgical patients after recovery from surgery (HR=0.4, CI 0.2–0.5). This may reflect the longer follow-up/survival time in this time category (median follow-up 25 months *vs* 3 months for non-surgical patients) where disease and treatment effects are likely to have less influence.

#### Chemotherapy

Chemotherapy patients exhibited the highest absolute VTE rate within this analysis (103.2 per 1000-person years during chemotherapy, CI 75.1, 141.8). If we investigate chemotherapy as a binary variable, that is, ever or never had chemotherapy, then the multivariable hazard ratio is 1.3 (CI 1.1–1.7). Although VTE risk in the time before chemotherapy is similar to that in non-chemotherapy patients (HR=1.1, CI 0.7–1.6), the risk doubled during chemotherapy (HR=2.4, CI 1.6–3.5) and then declined following cessation of chemotherapy, with a similar risk to baseline 2 months after chemotherapy ended.

#### Radiotherapy

Radiotherapy as defined by cancer registry data did not significantly affect the risk of VTE, though we were unable to assess the time-varying effect of radiotherapy as per surgery and chemotherapy due to low recording in the HES data.

### Survival of lung cancer patients

**Figure 2:**
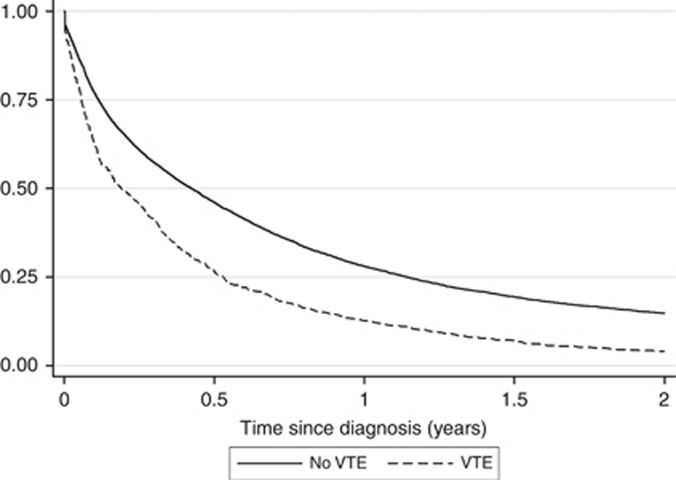
**Survival of lung cancer patients according to diagnosis of VTE**. To reduce the effect of immortal time bias, VTE was defined in a time-varying manner, whereby patients start in the ‘no VTE' group, and are switched to the ‘VTE' group after VTE diagnosis.

The effect of VTE diagnosis on survival was explored to determine its extent and whether any observed changes were independent or due to differences in patient mix. With VTE defined as a time-varying covariate, there is a clear increase in risk of death for patients with VTE (Figure 2 and Table 3) with an overall univariable HR of 1.7 (CI 1.5–1.8). Adjustment for the variables described in Table 2 only changes the HRs by a small amount (overall HR=1.5, CI 1.4–1.6), while the proportional hazards assumption was not broken (*P*=0.143). We found that there was a significant interaction between VTE and both histology (*P*=0.017) and diagnosis route (*P*<0.001) but not between VTE and stage (*P*=0.108). For histology, VTE appears to have little effect on survival in small cell patients, but is similar to the overall effect for other morphologies (Table 3). Additionally, VTE diagnosis seems to affect survival less in patients diagnosed through emergency admission. Additionally, PE and DVT were not found to affect survival in significantly different ways (PE HR: 1.6, CI 1.4–1.8; DVT 1.4, CI 1.2–1.5).

## Discussion

This study was able to determine the estimates of VTE risk for a wide range of potential risk factors related to lung cancer. Although the baseline rate of VTE remains higher in lung cancer than for most other cancer sites (Walker *et al*, 2012), we identified several groups of lung cancer patients with an exceptionally high rate of VTE. Figure 1 demonstrates that VTE events tend to cluster around the date of diagnosis and so we sought to determine the risk factors associated with these VTE events. Patient groups with the highest rates of VTE included those with adenocarcinoma, metastatic disease, emergency hospital admission around diagnosis and those receiving chemotherapy. From these data, it is apparent that grade, surgery, radiotherapy, comorbidity, BMI, age, smoking and platelet count are less important predictors of VTE in the lung cancer population. We also found that patients diagnosed with VTE had poorer survival on average.

It has been reported previously that adenocarcinoma patients may be at higher risk of VTE than other histologic subtypes (Blom *et al*, 2004; Tagalakis *et al*, 2007; Chew *et al*, 2008; Kadlec *et al*, 2014), indeed many other cancer sites with high incidence rates of VTE, such as pancreatic and bladder cancers are adenocarcinomas (Walker *et al*, 2012). Cancer stage is widely assumed to be a strong risk factor for VTE and our study agrees with that assumption, it does however show that much of the increased risk in people with metastatic disease may be due to confounding by other factors, with adjustment for surgery and chemotherapy accounting for the most of the difference between univariable and multivariable models. The increase in VTE rates in patients with emergency hospital admission around diagnosis is complex and likely to be a partial proxy indicator for late-stage disease, but also for performance status. Our data were mainly from an era with less emphasis on the use of thromboprophylaxis in medical in-patients, and the now widespread use of thromboprophylaxis assessment on admission may alter this finding.

The results we observed for platelet count in relation to VTE risk conflict somewhat with previous results. Although our model shows little effect, previous studies have demonstrated an association between high platelet counts and VTE (Zecchina *et al*, 2007). Although it is possible that there is no association with platelet count in this population, there are potential alternative explanations. It is likely that platelet count is confounded by other patient attributes, for example, stage or chemotherapy treatment that are imperfectly adjusted for, leading to residual confounding. Alternatively, patients with missing platelet counts may not be missing at random, meaning our results are liable to reflect the reasons that patients had their platelet levels recorded in these data as much as they are any direct effect raised platelet levels have on thrombosis risk. Age appeared to have a trend in the opposite direction to the overall cancer population (Walker *et al*, 2012), with lower rates in older age groups. This may be due to either less aggressive treatment in these higher age groups or alternatively reduced levels of investigation. This inverse trend has also been observed in other populations, for example in the California Cancer Registry (Chew *et al*, 2008) and Florida Medicaid Study (Huang *et al*, 2012).

The Californian study is the previous study most comparable to ours. They used a larger patient group, but reported on fewer potential risk factors than our study. Of those that were common to both studies, there was agreement in terms of the size and direction of the observed effects for age, cancer stage, pathology and surgery. They are, however, at odds in terms of comorbidity. Chew *et al* observed increasing VTE risk with increased comorbidity, whereas our study showed no such effect. This finding is replicated by Connolly *et al*, who also found that a higher comorbidity score was associated with greater VTE risk. Interestingly however, when comorbidities were examined individually by Connolly *et al*, only congestive heart failure was found to be associated. The study by Connolly *et al* also presents some interesting data on chemotherapy. It demonstrates that the majority of VTE events in the chemotherapy population occur in the first months after diagnosis, though does not distinguish chemotherapy from the period post chemotherapy as in our study. It is worth noting that in this and all similar studies, some of the increase in risk observed in chemotherapy patients could be due to ascertainment bias, that is, those receiving chemotherapy are more likely to have a CT scan which could identify occult PE.

Chemotherapy is increasingly considered as a powerful risk factor for VTE in the cancer population, to the extent that a well-validated risk prediction tool has been developed specifically for the population receiving chemotherapy (Khorana *et al*, 2008). Although this model takes cancer site into account, it is assumed that risk factors within the model exert a similar effect on VTE risk regardless of the cancer site. The pattern of VTE predictors in this study differs substantially from previous work carried out in the same data for colorectal cancer (Walker *et al*, 2014), where notably age and surgery were much stronger predictors of VTE. Risk factors also differ in breast cancer in these data, where age and BMI are strong predictors, with chemotherapy by far the greatest risk factor (Walker *et al*, 2016). This highlights the possible benefit of developing individual risk scores for each cancer type.

Although we found that patients with a VTE diagnosis had overall poorer survival than those without, it is difficult to infer causality to this finding. It is possible that some of the deaths are directly attributable to VTE, though given that the additional risk of death remains throughout follow-up for patients with VTE it is also likely to be due to patient mix and residual confounding (e.g., those with a VTE are more likely to die due to generally more advanced disease). We also determined that the effect of VTE on survival was less in patients with small cell lung cancer and those diagnosed through emergency admission. This may be due to these patients having a higher baseline hazard of death, making the additional hazard added by VTE diagnosis is less important.

An important strength of this study is that we used routinely recorded data which, in contrast to many previous studies which, for example use only hospitalised patients, only chemotherapy patients (Khorana *et al*, 2008) or only non-small cell patients (Tagalakis *et al*, 2007), should contain a representative sample of the whole lung cancer population. Unlike previous research, we assessed both surgery and chemotherapy in a time-dependent manner so that rates of VTE can be compared before, during and after therapy within the same group of patients.

Study limitations include missing data, on cancer stage especially, which undoubtedly reduced the power of our study in examining some risk factors, as well as the ability to adjust for confounding. Additionally, the level of missing data for stage particularly made it difficult to use imputation to analyse the data. Although our overall sample size was large, smaller patient subgroups presented limited ability to determine the detailed effects of risk factors, such as those undergoing surgery. It is possible that risk factors such as BMI would have shown more consistent trends if the highest and lowest categories had not been limited by small patient and event numbers. Another limitation is that radiotherapy recording within our database was limited to a single binary indicator variable. The implications of this are that we may have under-reported the level of radiotherapy within the population, and that we could not investigate time-varying effects of radiotherapy. Similarly, for patients who received outpatient chemotherapy while we were able to identify them as a separate risk group, we could not assess how their risk changed in relation to specific time intervals following chemotherapy.

We were unable to measure the effect of performance status on the risk of VTE, which is considered as an important risk factor for VTE as immobility; however, we did include a number of variables, such as route of diagnosis, stage and comorbidity, which are likely partial proxy indicators for performance status. Conversely, it is not known in this study whether route of diagnosis is a risk factor in its own right, or merely a proxy indicator for disease stage and performance status. It is also possible that a part of the obtained results are due to the measured risk factors being a marker for increased imaging, which we were not able to measure. For example, patients undergoing chemotherapy are arguably more likely to undergo staging CT scans, which might detect VTE events that are not clinically significant.

The clinical benefits of knowing which patients are most at risk of VTE and when this risk is highest may arise from knowing when to give thromboprophylaxis. Our stratification of VTE risk allows targeting of patients with exceptionally high risk, potentially preventing cases of VTE, which is in itself desirable given the potential reduction in morbidity. Conversely, there are groups of patients who are at a low risk of VTE, where thromboprophylaxis would cause a net harm due to side effects including bleeding and the inconvenience of a daily heparin injection. Although determination of the threshold above which the benefits of prophylaxis may outweigh the harms is beyond the scope of the present work, the absolute risks presented here are likely to help in the planning of future prophylaxis clinical trials where patients are selected for inclusion on the basis of their underlying risk.

Our study demonstrates that despite adjusting for multiple variables, patients who have a VTE event have a reduced survival in comparison with those who do not. This could be explained partially by increased deaths directly attributable to VTE, but could also be due to residual confounding (e.g., by disease severity). Though the recent trials testing the use of thromboprophylaxis in cancer patients have observed little corresponding survival benefit, they have been carried out in relatively large, roughly aggregated groups of patients, such as metastatic cancer patients (Verso *et al*, 2010) or chemotherapy patients (Agnelli *et al*, 2012). It is therefore possible that the improved targeting of thromboprophylaxis facilitated by this and similar studies will allow the potential survival benefits of preventing VTE events to be realised.

We have highlighted that despite risk of VTE being high in the whole lung cancer population, there are groups of patients where particular attention must be paid, namely those with metastatic disease and those undergoing chemotherapy. There are also groups that have much lower risk, such as long-term survivors not receiving treatment. It is imperative that future randomised trials use these and similar data when selecting patients for thromboprophylaxis.

## Figures and Tables

**Table 1:** Characteristics of lung cancer patients with and without VTE

	**No VTE**	**%**	**VTE**	**%**
Total	10 234		364	
**Histology**				
Small cell	1259	12.3	33	9.1
Squamous cell	2454	24.0	74	20.3
Adenocarcinoma	1532	15.0	100	27.5
Other	1177	11.5	43	11.8
Unknown	3812	37.2	114	31.3
**Cancer stage**				
Local disease	745	7.3	29	8.0
Regional disease	394	3.8	19	5.2
Distant metastases	1902	18.6	73	20.1
Unknown	7193	70.3	243	66.8
**Route of diagnosis**				
Elective hospitalisation	4944	48.3	227	62.4
Emergency (A&E)	1805	17.6	52	14.3
Emergency (other)	2024	19.8	54	14.8
No diagnostic hospitalisation	1461	14.3	31	8.5
**Age bands**				
<50	345	3.4	20	5.5
50–59	1291	12.6	74	20.3
60–69	2727	26.6	131	36.0
70–79	3838	37.5	113	31.0
⩾80	2033	19.9	26	7.1
**Comorbidities**				
0	3625	35.4	136	37.4
1–3	5557	54.3	188	51.6
⩾4	1052	10.3	40	11.0
**Smoking**				
No	7669	74.9	278	76.4
Yes	2565	25.1	86	23.6
**BMI**				
Underweight	331	3.2	9	2.5
Ideal	2378	23.2	87	23.9
Overweight	1739	17.0	86	23.6
Obese	503	4.9	23	6.3
Morbidly obese	132	1.3	4	1.1
Missing	5151	50.3	155	42.6
**Surgery**				
No	9155	89.5	295	81.0
Yes	1079	10.5	69	19.0
**Chemotherapy**				
No	7644	74.7	202	55.5
Yes	2478	24.3	162	44.5
**Radiotherapy**				
No	7096	69.3	226	62.1
Yes	3165	30.9	138	37.9

Abbreviations: BMI=body mass index; VTE=venous thromboembolism.

**Table 2:** Rates of VTE in relation to potential risk factors

	**Absolute rates (per 1000 person-years)**				**Univariate Cox model**		**Multivariate Cox model**^a^	
	**Events**	**Time**	**Rate**	**95% CI**	**HR**	**95% CI**	**HR**	**95% CI**
**Histology**								
Small cell	33	1.0	31.7	22.5–44.5	1.2	0.8–1.9	0.7	0.4–1.0
Squamous cell	74	3.0	24.9	19.8–31.2	Reference		Reference	
Adenocarcinoma	100	1.9	51.4	42.3–62.6	2.1	1.5–2.8	1.9	1.4–2.6
Other	43	1.2	36.3	27.0–49.0	1.5	1.0–2.1	1.3	0.8–1.8
Unknown	114	2.1	53.4	44.5–64.2	2.1	1.5–2.8	1.8	1.3–2.4
**Stage**								
Localised	29	1.4	20.9	14.5–30.1	Reference		Reference	
Regional	19	0.6	31.7	20.2–49.7	1.5	0.9–2.7	1.3	0.7–2.3
Distant Metastases	73	0.9	81.7	65.0–102.8	3.6	2.4–5.6	1.8	1.1–2.9
Unknown	243	6.4	38.0	33.5–43.1	1.8	1.2–2.6	1.3	0.8–1.9
**Grade**								
Well differentiated	7	0.3	24.0	11.4–50.3	Reference		Reference	
Moderately well differentiated	40	1.5	26.8	19.7–36.6	1.1	0.5–2.4	1.3	0.6–2.9
Poorly differentiated	91	2.1	43.3	35.2–53.1	1.7	0.8–3.7	1.6	0.7–3.4
Unknown	226	5.4	41.9	36.8–47.7	1.7	0.8–3.5	1.3	0.6–2.9
**Route of diagnosis**								
Elective hospitalisation	227	6.7	33.8	29.6–38.4	Reference		Reference	
Emergency hospitalisation	106	1.5	71.8	59.4–86.9	2.0	1.6–2.6	1.6	1.3–2.1
No in-patient hospitalisation	31	1.1	28.7	20.2–40.8	0.8	0.6–1.2	0.7	0.5–1.0
**Surgery**								
No surgery	295	5.8	50.7	45.2–56.8	Reference		Reference	
Before surgery	5	0.2	30.9	12.9–74.2	0.6	0.2–1.5	0.6	0.3–1.6
During surgery hospitalisation	3	0.0	101.0	32.6–313.2	1.9	0.6–6.0	2.0	0.6–6.4
1 month after	4	0.1	45.7	17.1–121.7	0.9	0.4–2.5	1.0	0.4–2.7
2 months after	3	0.1	37.1	12.0–115.1	0.7	0.2–2.3	0.8	0.2–2.4
3 months after	6	0.1	78.0	35.1–173.7	1.5	0.7–3.4	1.5	0.7–3.4
Subsequent time^b^	48	3.0	15.8	11.9–21.0	0.3	0.2–0.4	0.4	0.2–0.5
**Chemotherapy**								
No chemotherapy	202	6.2	32.5	28.3–37.3	Reference		Reference	
Before chemotherapy	24	0.6	42.6	28.5–63.5	1.3	0.8–1.9	1.1	0.7–1.6
During chemotherapy	38	0.4	103.2	75.1–141.8	3.1	2.2–4.4	2.4	1.6–3.5
1 month after	10	0.1	86.8	46.7–161.2	2.6	1.4–4.9	2.0	1.0–3.8
2 months after	5	0.1	53.6	22.3–128.7	1.5	0.6–3.8	1.2	0.5–3.0
3 months after	0	0.1	0.0		—		—	
Subsequent time^b^	30	1.0	28.7	20.1–41.1	0.8	0.6–1.2	0.7	0.5–1.1
Outpatient chemotherapy^c^	55	0.8	69.1	53.1–90.0	2.1	1.6–2.8	1.7	1.2–2.3
**Radiotherapy**								
No	226	6.1	36.9	32.4–42.1	Reference		Reference	
Yes	138	3.2	43.7	37.0–51.6	1.1	0.9–1.4	0.9	0.7–1.1
**Comorbidity**								
0	136	3.4	40.5	34.2–47.9	Reference		Reference	
1–3	188	4.9	38.1	33.1–44.0	1.0	0.8–1.2	1.0	0.8–1.3
>3	40	1.0	40.3	29.5–54.9	1.1	0.8–1.5	1.1	0.7–1.6
**BMI**								
Underweight	9	0.3	30.3	15.8–58.2	0.8	0.4–1.5	0.7	0.4–1.5
Ideal	87	2.3	37.8	30.6–46.6	Reference		Reference	
Overweight	86	1.9	45.0	36.4–55.6	1.2	0.9–1.6	1.4	1.0–1.8
Obese	23	0.5	44.2	29.4–66.5	1.2	0.8–1.9	1.2	0.8–2.0
Morbidly obese	4	0.2	24.9	9.3–66.2	0.7	0.2–1.8	0.7	0.2–1.9
Missing	155	4.1	37.9	32.4–44.4	0.9	0.7–1.2	1.0	0.8–1.3
**Age**								
<50	20	0.6	35.7	23.1–55.4	Reference		Reference	
50–60	74	1.6	46.0	36.7–57.8	1.3	0.8–2.1	1.3	0.8–2.1
60–70	131	3.0	43.1	36.3–51.1	1.2	0.8–1.9	1.2	0.8–2.0
70–80	113	3.1	36.3	30.2–43.6	1.0	0.6–1.6	0.9	0.6–1.5
>80	26	1.0	27.2	18.5–40.0	0.7	0.4–1.3	0.6	0.3–1.0
**Smoking**								
No	278	7.6	36.6	32.6–41.2	Reference		Reference	
Yes	86	1.7	50.7	41.0–62.6	1.4	1.1–1.7	1.2	0.9–1.5
**Platelet count**								
Low (<140)	14	0.2	59.4	35.2–100.3	1.6	0.9–2.8	1.5	0.9–2.6
Normal (140–400)	195	5.5	35.3	30.7–40.6	Reference		Reference	
High (>400)	50	1.4	36.8	27.9–48.5	1.0	0.7–1.4	0.9	0.6–1.2
No count	105	2.2	48.5	40.0–58.7	1.3	1.0–1.6	1.1	0.8–1.4

Abbreviations: BMI=body mass index; CI=confidence interval; VTE=venous thromboembolism.

a Hazard ratios adjusted for all other variables in table.

b Subsequent time refers to the time after the procedure until the completion of follow-up.

c Outpatient chemotherapy refers to those patients who were recorded as having chemotherapy in the cancer registry, but did not have a record of this in the in-patient hospital records.

**Table 3:** Survival of patients with VTE events

	**HR**	**95% CI**
All patients	1.50	1.37–1.63
**Histology**		
Small cell	1.02	0.76–1.35
Squamous cell	1.52	1.24–1.87
Adenocarcinoma	1.63	1.36–1.95
Other	1.44	1.14–1.84
Unknown	1.60	1.38–1.85
**Stage**		
Localised	2.12	1.50–3.02
Regional	1.47	0.93–2.34
Distant metastases	1.84	1.52–2.25
Unknown	1.42	1.28–1.58
**Route of diagnosis**		
Elective hospitalisation	1.68	1.49–1.90
Emergency hospitalisation	1.30	1.14–1.49
No in-patient hospitalisation	1.65	1.16–2.35

Abbreviations: BMI=body mass index; CI=confidence interval; VTE=venous thromboembolism. Multivariable hazard ratios for survival of patients with VTE events (reference group non-VTE patients) with VTE defined in a time-varying manner. Table is stratified by patient characteristics. Multivariable Cox model adjusted for histology, stage, grade, diagnosis route, surgery, chemotherapy, radiotherapy, BMI, age and comorbidity.
